# Novel tumor suppressor microRNA at frequently deleted chromosomal region 8p21 regulates Epidermal Growth Factor Receptor in prostate cancer

**DOI:** 10.18632/oncotarget.11865

**Published:** 2016-09-06

**Authors:** Nathan Bucay, Kirandeep Sekhon, Shahana Majid, Soichiro Yamamura, Varahram Shahryari, Z. Laura Tabatabai, Kirsten Greene, Yuichiro Tanaka, Rajvir Dahiya, Guoren Deng, Sharanjot Saini

**Affiliations:** ^1^ Department of Urology, Veterans Affairs Medical Center, San Francisco and University of California San Francisco, CA, USA

**Keywords:** prostate cancer, miR-3622b, EGFR, chr8p21, tumor suppressor

## Abstract

Genomic loss of chromosome (chr) 8p21 region, containing prostate-specific *NKX3.1* gene, is a frequent alteration of the prostate cancer (PCa) oncogenome. We propose a novel, paradigm shifting hypothesis that this frequently deleted locus is also associated with a cluster of microRNA genes- miR-3622a/b- that are lost in PCa and play an important mechanistic role in progression and metastasis. In this study, we demonstrate the role of miR-3622b in prostate cancer. Expression analyses in a cohort of PCa clinical specimens and cell lines show that miR-3622b expression is frequently lost in prostate cancer. Low miR-3622b expression was found to be associated with tumor progression and poor biochemical recurrence-free survival. Further, our analyses suggest that miR-3622b expression is a promising prostate cancer diagnostic biomarker that exhibits 100% specificity and 66% sensitivity. Restoration of miR-3622b expression in PCa cell lines led to reduced cellular viability, proliferation, invasiveness, migration and increased apoptosis. miR-3622b overexpression *in vivo* induced regression of established prostate tumor xenografts pointing to its therapeutic potential. Further, we found that miR-3622b directly represses Epidermal Growth Factor Receptor (EGFR). In conclusion, our study suggests that miR-3622b plays a tumor suppressive role and is frequently downregulated in prostate cancer, leading to EGFR upregulation. Importantly, miR-3622b has associated diagnostic, prognostic and therapeutic potential. Considering the association of chr8p21 loss with poor prognosis, our findings are highly significant and support a novel concept that associates a long standing observation of frequent loss of a chromosomal region with a novel miRNA in prostate cancer.

## INTRODUCTION

Prostate cancer (PCa) is the most common male malignancy worldwide and a leading cause of cancer related mortality amongst men [1, 2] with an estimated 26,120 deaths predicted for 2016 within the US [2]. Prostate tumors are often indolent and can be effectively managed by active surveillance and watchful waiting [3, 4]. However, a significant proportion of prostate tumors are likely to progress, often metastasizing to bone and other organs causing significant morbidity and mortality [5]. Though novel therapies with marginal survival benefits have been developed for advanced disease [6, 7], its clinical management remains challenging. Also, PCa is associated with high rates of recurrence, with approximately 40% of men with localized PCa suffering from relapse after initial therapy [8] as monitored by rising PSA and tumor progression to a hormone refractory/castration resistant stage [1, 9] that is essentially untreatable [1, 10]. A major challenge is the elucidation of underlying molecular pathways of PCa progression, recurrence and metastasis that holds enormous potential towards the design of effective therapeutic strategies for better clinical management of the disease.

The prostate cancer genome is typically characterized by frequent deletion of chromosome 8p (chr8p) region in 40%-70% cases [11–17]. Taylor *et al.* performed large scale integrative analyses of transcriptomes and copy-number alterations (CNAs) in prostate adenocarcinomas and reported chr8p loss as the most frequent genomic alteration [17]. In a recent *in silico* meta analyses of PCa genomic data from 662 patients (546 primary, 116 advanced tumors), chr8p deletion was reported as the most recurrent CNA [18]. Chr8p losses were reported in 55.7% cases of localized and 90.5% cases of advanced PCa [18]. Association of advanced PCa with a significantly higher deletion frequency of this region [19] suggests its role in disease progression. The minimal region of deletion occurs at chr8p21 [18], which contains prostate-specific *NKX3.1* tumor suppressor gene [20]. Also, studies have shown high rate of loss of heterozygosity (LOH) at chr8p21 subregion [21, 22] that has been associated with prostate-specific homeobox gene *NKX3.1* [20]. Recent genomic studies suggest the possibility of alternate tumor suppressor genes within this region, apart from *NKX3.1* [17]. However, the genes within this region are yet to be fully characterized. We propose a novel, paradigm shifting hypothesis that this frequently deleted locus is associated with a cluster of microRNA genes- miR-3622a/b- that are lost in PCa and play an important mechanistic role in PCa progression and metastasis. MicroRNAs (miRNAs) are endogenous small RNAs that suppress gene expression post transcriptionally via sequence-specific interactions with the 3'- untranslated regions (UTRs) of cognate mRNA targets [23], and are often located in fragile chromosomal regions involved in cancers [24]. We previously demonstrated that miR-3622a located on frequently deleted chr8p21 region plays a crucial role in PCa epithelial-to-mesenchymal transition (EMT), progression and metastasis by direct targeting of ZEB1 and SNAI2 (Bucay *et al*., manuscript under consideration). Here we examined the role of another member of the cluster- miR-3622b- in PCa and show its crucial tumor suppressive role. miR-3622b is a recently discovered miRNA gene [25] that has not been studied. Expression analyses in a cohort of PCa clinical specimens showed that miR-3622b expression is frequently lost in PCa and correlated with poor recurrence-free survival outcome and tumor progression. Our *in vitro* and *in vivo* studies support a tumor suppressive role of miR-3622b in PCa, mediating its effects largely by directly repressing Epidermal Growth Factor Receptor (EGFR). Thus, our data support a novel concept, linking the long standing observation of frequent loss of chr8p21 in PCa, with the loss of a novel miRNA gene within this region.

## RESULTS

### MicroRNA-3622b located in frequently deleted chr8p21 region is underexpressed in prostate cancer

**Figure 1:**
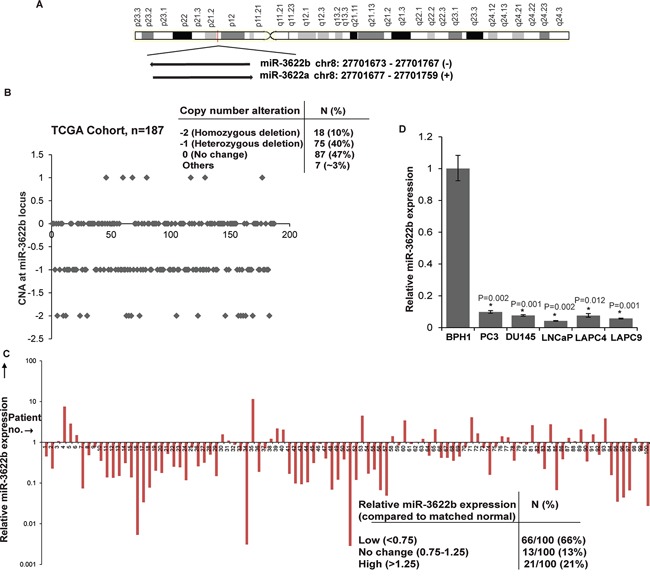
MicroRNA-3622b located in frequently deleted chr8p21 region is under expressed in prostate cancer **A.** Schematic representation of chr8p21 region highlighting the location of miR-3622a/b cluster. miR-3622b is located at position 27701673-27701767 on the antisense strand. **B.** CNAs at miR-3622b locus in the TCGA cohort of prostate adenocarcinomas. **C.** miR-3622b expression levels in PCa specimens relative to matched adjacent normals as assessed by real-time PCR. **D.** Relative miR-3622b expression levels in immortalized non-malignant cell line (BPH1) and PCa cell lines as assessed by RT-PCR. Data were normalized to RNU48 control. (*P< .05).

Along with the *NKX3.1* gene [25, 26], frequently lost Chr8p21 region contains a cluster of miRNA genes- miR-3622a and miR-3622b (Figure 1A). We analyzed CNAs at the miR-3622b locus in prostate adenocarcinomas in The Cancer Genome Atlas (TCGA) dataset (n=187) [27, 28]. Similar to miR-3622a, miR-3622b locus was deleted either homozygously or heterozygously in ~ 50% cases suggesting that prostate tumors are associated with a frequent genomic deletion of this miRNA (Figure 1B). Based on this, we hypothesized that miR-3622b expression is lost in PCa and may underlie prostate carcinogenesis. To test our hypothesis, we first performed miR-3622b-5p (referred to as miR-3622b) expression profiling in a large cohort of PCa clinical specimens (n=100) and matched adjacent normal regions by real-time PCR (Figure 1C). miR-3622b expression was significantly downregulated in ~66% of PCa cases (Wilcoxon Signed Rank test P-value < 0.0001). 13% of PCa cases showed no change and ~21% cases exhibited high expression. Patients' demographics and clinicopathological characteristics are summarized in Table 1. We also analyzed miR-3622b expression in prostate cell lines (Figure 1D) showing that its expression is specifically attenuated in PCa cell lines (PC3, DU145, LNCaP, LAPC4, LAPC9) compared to BPH1 cell line. These data point to the widespread attenuated expression of miR-3622b in PCa.

**Table 1:** Clinicopathologic characteristics of prostate cancer patients (N = 100)

Characteristics	Number of patients N(%)
Age, Years^*^	
Mean	62.5
Median	62
Range	47-79
T-stage^**^	
pT2	3 (3)
pT2a	12 (12)
pT2b	15 (15)
pT2c	34 (34)
pT3	1 (1)
pT3a	15 (15)
pT3b	5 (5)
pT4	1 (1)
Gleason Score^***^	
6	53 (53)
7	33 (33)
8-10	10 (10)
PSA^****^	
Median	4.7
<4.7	32 (32)
>4.7	59 (59)
**PSA failure**	35
**N-stage**	
N0/NX	100 (100)
**M-stage**	
M0/MX	100 (100)
**Pathological diagnosis**	
Adenocarcinoma	100 (100)

* No information on age for 3 cases

** Tumor stage unknown for 14 cases

*** Gleason score unknown for 4 cases

**** PSA values not known for 9 cases

Clinicopathological characteristics of prostate cancer clinical tissues used for real-time PCR analysis of miR-3622b expression. T-stage refers to the pathological stage of the primary tumor, ranging from pT2 to pT4. N-stage indicates if the cancer has spread to nearby regional lymph nodes where NX refer to cases with lymph nodes not assessed and N0 denotes cases with tumor not spread to nearby lymph nodes. M-stage refers to metastasis to distant organs wherein M0 denotes absence of metastasis and MX denotes cases with metastasis not assessed. Gleason score indicates the grade or differentiation of primary tumors ranging from well-differentiated or low grade (cancers with a Gleason score of 6) to moderately-differentiated (Gleason score 7) or high grade or poorly differentiated (Gleason scores of 8-10) tumors. Additional parameters include serum PSA levels at the time of diagnosis and PSA failure/biochemical recurrence.

### Low miR-3622b expression is associated with tumor progression and biochemical recurrence in prostate cancer

**Figure 2:**
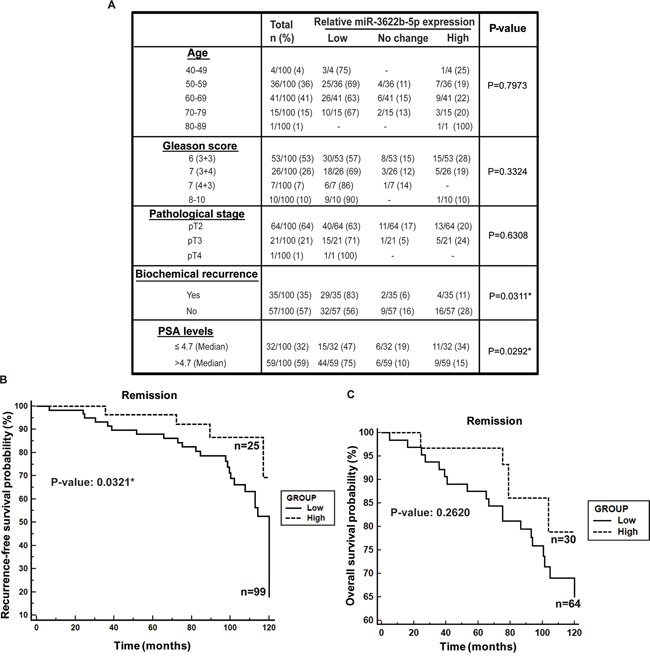
Low miR-3622b expression is associated with biochemical recurrence in prostate cancer **A.** Correlation of miR-3622b expression with clinicopathological characteristics of prostate cancer patient cohort. P-values based on a Chi square test. **B.** Kaplan-Meier survival analysis for recurrence-free survival (RFS) of PCa patients, stratified based on miR-3622b levels. P-value based on a log rank test. **C.** Kaplan-Meier analyses for overall survival of PCa patients, stratified based on miR-3622b levels. P-value based on a log rank test.

In view of the observed widespread low miR-3622b expression in PCa clinical tissues, we evaluated the correlation of miR-3622b expression with clinicopathological parameters of the disease (Figure 2A). While no correlation was observed between miR-3622b expression and age, low miR-3622b expression was observed in 57% cases of Gleason 6, 69% cases of Gleason 7 (3+4), 86% of Gleason 7 (4+3) and 90% cases of Gleason 8-10. Similarly, decreased miR-3622b expression was observed in 63% of cases of pathological stage pT2, 71% of cases of pT3 and 100% of pT4 cases. This trend suggests that miR-3622b expression is progressively downregulated in tumors with higher Gleason grade and stage though this correlation failed to reach statistical significance. Interestingly, statistically significant correlation was observed between low miR-3622b expression and biochemical recurrence (BCR). Kaplan-Meier survival analysis for recurrence-free survival (RFS) (Figure 2B) showed that cases with low miR-3622b expression had a significantly lower recurrence-free survival probability than those with high expression (P=0.0321). Also, cases with low miR-3622b expression had lower overall survival probability (Figure 2C) as compared to high expressors though this result was statistically insignificant with the present cohort.

### Low miR-3622b expression is correlated with high serum PSA in prostate cancer

**Figure 3:**
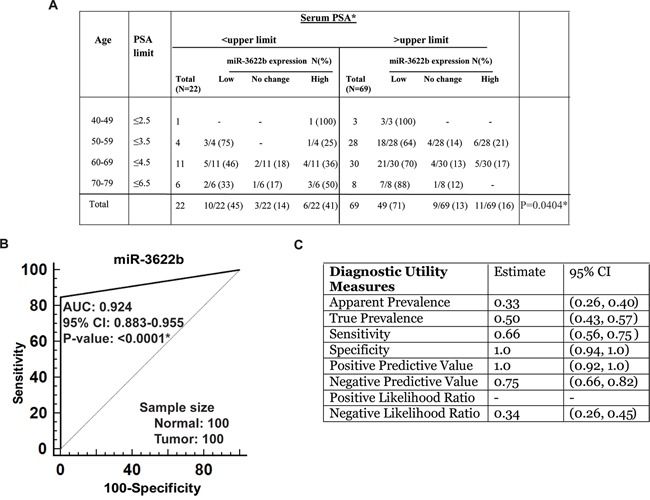
Potential diagnostic utility of miR-3622b expression in prostate cancer **A.** Correlation of miR-3622b expression with age-adjusted serum PSA levels in PCa patients. (* P< .05). **B.** ROC curve analysis showing the ability of miR-3622b expression to discriminate between malignant and non-malignant cases. **C.** Diagnostic utility measures of miR-3622a as a prostate cancer biomarker. (* P< .05).

We observed significant inverse correlation between miR-3622b expression and serum PSA levels (P=0.0292) (Figure 2A). Low miR-3622b expression was observed in 47% of PCa cases with low PSA and in 75% of cases with high PSA levels. We also analyzed this correlation using age-adjusted PSA values (Figure 3A) and found that miR-3622b expression is statistically correlated with age-adjusted PSA levels (P= 0.0404). In view of these results, we performed ROC analyses to test the diagnostic potential of miR-3622b expression (Figure 3B). This analyses showed that miR-3622b expression can be a single significant parameter to discriminate between normal and tumor tissues with AUC of 0.924 (95% CI: 0.883-0.955, P<0.0001). The discriminatory ability of miR-3622b as a diagnostic classifier was further characterized. Our analyses showed that miR-3622b as a diagnostic biomarker exhibits a specificity of 1, sensitivity of 0.66, positive predictive value of 1.0 and negative predictive value of 0.75 (Figure 3C). Overall, our analyses suggest that miR-3622b has significant potential as a diagnostic biomarker for PCa.

### miR-3622b overexpression suppresses tumorigenicity *in vitro* in prostate cancer cell lines

**Figure 4:**
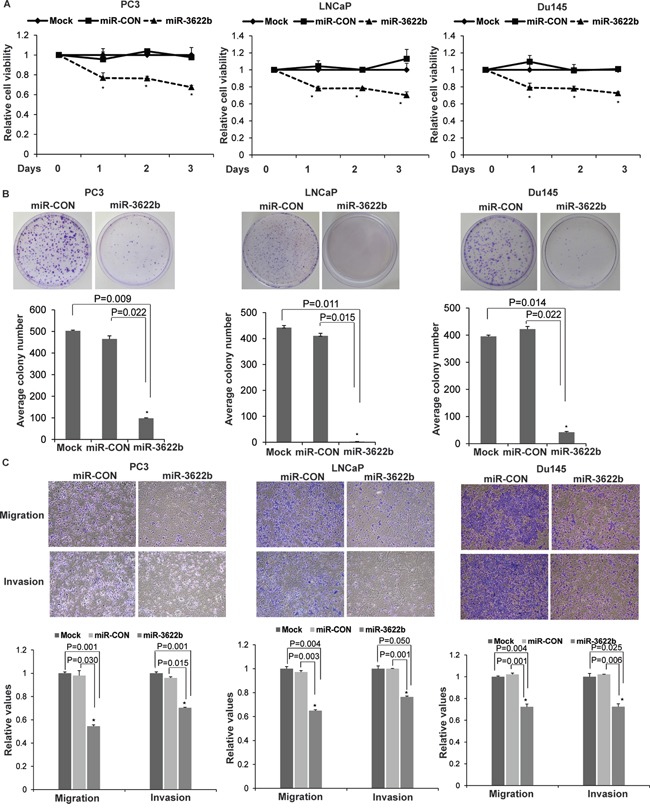
miR-3622b overexpression suppresses tumorigenicity *in vitro* in prostate cancer cell lines To assess the functional significance of miR-3622b, miR-3622b or a control miR (miR-CON) was overexpressed in PCa cell lines (PC3, LNCaP, Du145) by transient transfections followed by functional assays 72 hrs post-transfection (* P< .05). **A.** Cell viability assays **B.** Colony formation assays **C.** Transwell migration and invasion assays in PC3/ LNCaP/ Du145 cells transfected with mock/ miR-CON/ miR-3622b.

In view of low miR-3622b expression levels in PCa clinical specimens and PCa cell lines, we evaluated the tumor suppressive potential of miR-3622b overexpression *in vitro* and *in vivo*. miR-3622b overexpression (Supplementary Figure S1) in PCa cell lines PC3, LNCaP and Du145 led to reduced cellular viability (Figure 4A) as compared to control miRNA (miR-CON) transfected cells. miR-3622b overexpression decreased the clonogenicity (Figure 4B), migration and invasiveness (Figure 4C) of PC3/LNCaP/Du145 cells. These observations demonstrate that miR-3622b overexpression suppresses the *in vitro* attributes of tumorigenicity in PCa cell lines.

### miR-3622b overexpression induces apoptosis in PCa cell lines

**Figure 5:**
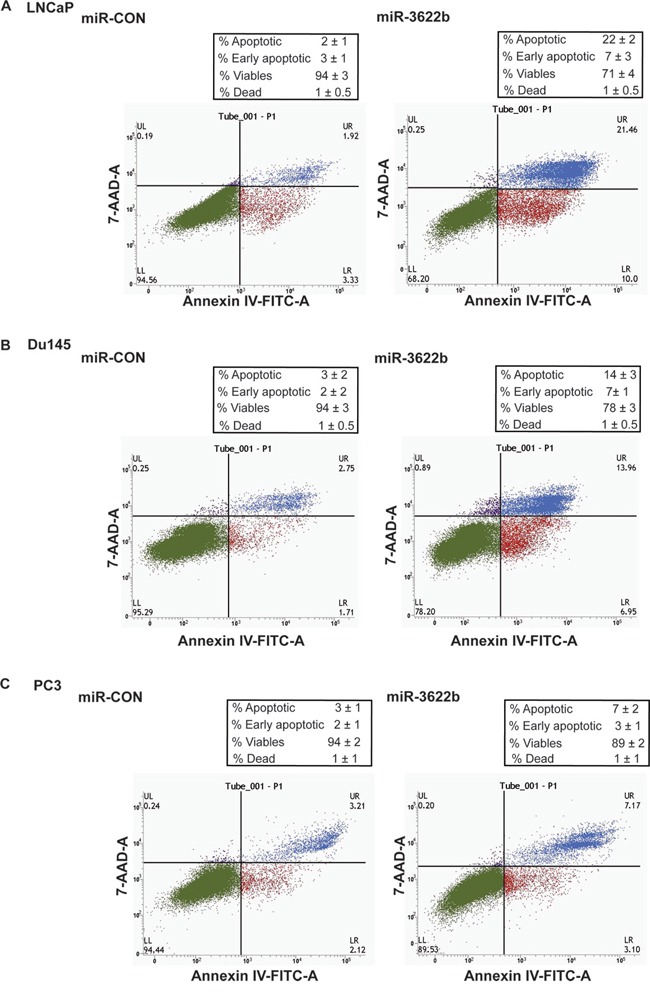
miR-3622b overexpression induces apoptosis in prostate cancer cell lines Flow cytometric analysis of Annexin-V-FITC-7-AAD stained **A.** LNCaP, **B.** Du145 and **C.** PC3 transfected with miR-CON/ miR-3622b.

miR-3622b overexpression led to marked morphological changes in PCa cell lines with the cells transitioning from an elongated, spindle shaped morphology to rounded, apoptotic cells suggesting that miR-3622b overexpression leads to induction of apoptosis. To confirm this, we performed flow cytometric analysis of Annexin-V-FITC-7-AAD stained LNCaP/Du145/PC3 cells transfected with miR-CON/miR-3622b (Figure 5A-5C). It was observed that the average apoptotic cell fractions (early apoptotic + apoptotic) were significantly (P=0.026) increased with a concomitant decrease in the viable cell populations upon miR-3622b overexpression in PCa cell lines compared to miR-CON transfected cells. These analyses suggest that miR-3622b affects apoptotic pathways in PCa and plays a pro-apoptotic role. In line with these data, we found that miR-3622b overexpresssion induces poly-ADP-ribose polymerase (PARP) cleavage in PCa cell lines (Supplementary Figure S2).

### Intratumoral delivery of miR-3622b leads to tumor regression in PCa xenografts

**Figure 6:**
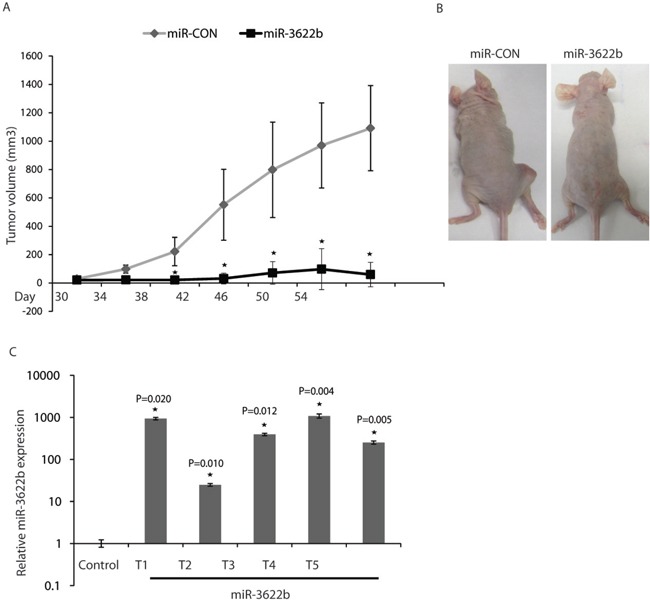
Intratumoral delivery of miR-3622b leads to tumor regression in PCa xenografts PC3 cells were subcutaneously injected into nude mice and maintained until solid, palpable tumors (Day 30), following which control miRNA/ miR-3622b mimics were injected intratumorally at periodic intervals (indicated) and tumor growth was monitored. (* P< .05). **A.** Tumor volumes following miR-CON/miR-3622b administration at the indicated time points. **B.** Representative images of mice from the two groups on day 54 are shown. **C.** Relative miR-3622b expression in PCa xenografts as assessed by real-time PCR.

In view of the tumor suppressive effects of miR-3622b overexpression observed *in vitro*, we examined the therapeutic potential of miR-3622b in a PCa xenograft mouse model (Figure 6). PC3 cells were subcutaneously injected into nude mice and maintained until solid, palpable tumors were formed. Control miRNA or miR-3622b mimics were injected intratumorally at periodic intervals and tumor growth was monitored regularly. Interestingly, we observed a significant tumor growth inhibition in mice injected with miR-3622b mimics supporting the tumor suppressive effects of this miRNA in PCa (Figure 6A-6B). To confirm that the observed tumor regression was correlated to miR-3622b delivery, we harvested the tumors from control or miR-3622b (n=5) groups followed by RNA extractions and real time PCR based analyses of miR-3622b levels (Figure 6C). miR-3622b expression was significantly higher in tumors injected with synthetic miR-3622b versus control tumors.

### miR-3622b targets Epidermal Growth Factor Receptor in prostate cancer

**Figure 7:**
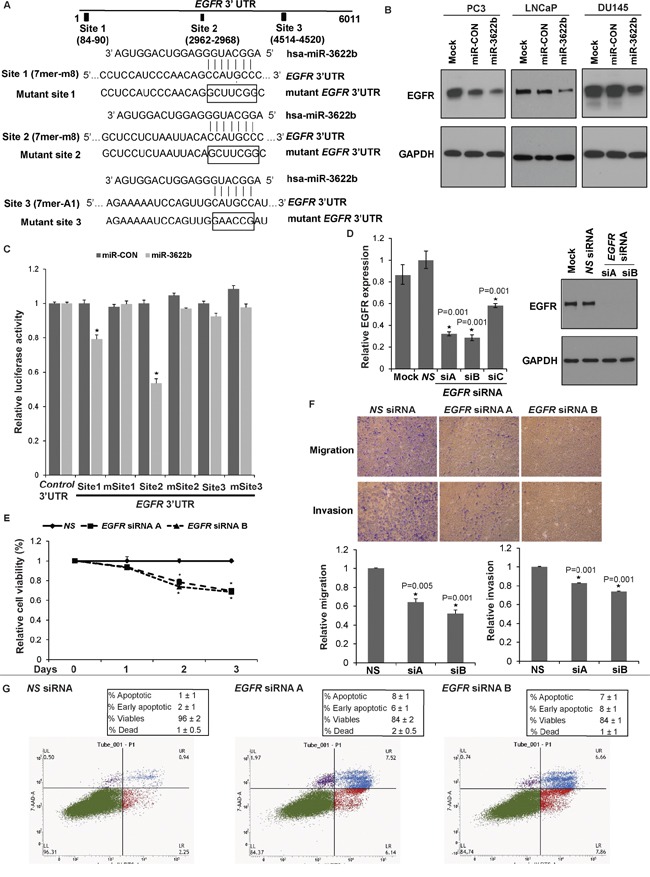
miR-3622b targets Epidermal Growth Factor Receptor in prostate cancer **A.** Immunoblots of endogenous EGFR and BMI1 in PC3 (left panels), LNCaP (middle panels) and Du145 cells (right panels) transfected with mock/miR-CON/miR-3622b. GAPDH was used a loading control. **B.** Schematic representation of the *EGFR* 3'-UTR showing the putative miR-3622b binding sites. Respective mutant *EGFR* sites are represented below. **C.** Luciferase reporter assays with the indicated wt and mutant *EGFR* 3' UTR constructs or control luciferase construct co-transfected with miR-CON/ miR-3622b in PC3 cells. Firefly luciferase values were normalized to Renilla luciferase activity and plotted as relative luciferase activity (* P< .05). **D.** LNCaP cells were transfected with three sets of siRNAs (siRNA-A, siRNA-B, siRNA-C) against *EGFR* or a nonspecific (*NS*) control siRNA/mock transfected for 72 h (*P< .05) followed by functional assays. Left panel: Real time PCR analyses of relative *EGFR* expression. Data were normalized to *GAPDH* control. siRNA-A and siRNA-B were effective in knocking down *EGFR* expression by ~70% as compared to *NS* and were used in subsequent experiments. Right panel: Immunoblot analyses for EGFR protein expression after *NS/EGFR* siRNA transfections. GAPDH was used as a loading control. **E.** Cellular viability assay, **F.** Transwell migration and invasion assay after *NS/EGFR* siRNA- A and siRNA-B transfections. **G.** Apoptosis assay upon *NS* siRNA (left panel) or *EGFR* siRNA-A (middle panel) or siRNA-B (right panel) transfections.

To identify miR-3622b targets, we performed *in silico* analyses followed by Western blotting of potential miR-3622b targets. Our analyses identified that miR-3622b represses Epidermal Growth Factor Receptor (EGFR) and polycomb repressor BMI1 in PCa cell lines (Figure 7A and Supplementary Figure S3). Immunoblotting of mock/miR-CON/miR-3622b overexpressing PC3/LNCaP/Du145 cells confirmed that miR-3622b represses EGFR protein levels in all cell lines (Figure 7A). Also, miR-3622b inhibited the expression of BMI1, primarily in LNCaP/Du145 cell lines. The 3' UTR region of *EGFR* possess three potential miR-3622b binding sites while that of *BMI1* has 1 site (Figure 7B and Supplementary Figure S3A). Due to the modest effects on BMI1 expression observed in PC3 cells, we focused on EGFR as a target. To verify that the repressive effects on EGFR expression are as a result of direct interaction of miR-3622b with the corresponding microRNA binding sites within *EGFR* 3' UTR, we constructed luciferase reporter vectors with potential miR-3622b sites 1-3. *EGFR* 3' UTR possesses two 7mer-m8 sites and one 7mer-A1 site (Figure 7B) [29, 30]. To further validate these sites, we mutated the potential miR-3622b binding sites in *EGFR* 3' UTR as represented in Figure 7B. Luciferase reporter assays with wt and mutant *EGFR* 3'UTR/Control 3'UTR in miR-CON/miR-3622b transfected PC3 cells (Figure 7C) showed that there was a decrease in wild type *EGFR* luciferase reporter activity upon miR-3622b overexpression while the mutation of the miR-3622b binding sites prevented the repression of luciferase activity. Luciferase reporter activity with miR-3622b binding sites 1 and 2 showed a significant decrease while the observed repression with site 3 reporter construct was statistically insignificant suggesting that miR-3622b represses *EGFR* predominantly through its direct binding to two 7mer-m8 sites within its 3'UTR.

### *EGFR* knockdown partially phenocopies miR-3622b overexpression in LNCaP cells

To determine if EGFR represents a functionally relevant target of miR-3622b in PCa, we looked to see if *EGFR* knockdown functionally mimics the effects of miR-3622b overexpression in PCa. Towards this, we performed siRNA mediated-*EGFR* knockdown in LNCaP cells followed by functional assays (Figure 7E-7G). We initially tested three sets of siRNAs (siRNA-A, siRNA-B, siRNA-C) against *EGFR* and assessed the knockdown efficiency by RT-PCR analyses (Figure 7D, left panel). siRNA-A and siRNA-B were effective in knocking down *EGFR* expression by ~70% as compared to negative control scrambled siRNA (*NS*) and were used in subsequent experiments. Immunoblotting analyses confirmed knockdown of EGFR protein levels after siRNA-A and -B treatment (Figure 7D, right panel). *EGFR* inhibition led to decreased cellular viability (Figure 7E), migration and invasiveness of LNCaP cells (Figure 7F). Also, apoptotic cell fractions (early apoptotic + apoptotic) were significantly increased upon *EGFR* knockdown as compared to *NS* siRNA, an effect similar to that observed upon miR-3622b overexpression in LNCaP cells (Figure 7G). These results suggest that *EGFR* inhibition partially phenocopies the effects of miR-3622b overexpression in the LNCaP cell line.

## DISCUSSION

Loss of chromosome 8p [11–17]- particularly chr8p21 subregion- [21, 22] – has been consistently reported as a frequent alteration of the PCa genome that has been associated with the loss of tumor suppressors such as prostate-specific *NKX3.1* gene [20]. Our study uncovers the role of a novel alternative tumor suppressor miRNA gene- miR-3622b- in this region that plays a crucial role in PCa by regulating EGFR. miR-3622b is a member of the miR-3622a/b cluster located within the chr8p21 region [25]. Our earlier study demonstrated an important role of miR-3622a in PCa EMT, progression and metastasis (Bucay *et al*., manuscript under consideration). Studies show a significant increase in chr8p deletions with tumor progression and poor prognosis in PCa [31, 32], pointing to its important mechanistic role in PCa progression. Our studies lend support to this notion. Also, it has been suggested that copy number alterations frequently lead to loss of multiple genes that may cooperate to produce more aggressive disease [33]. Our present study, in conjunction with our earlier study on miR-3622a, support our hypothesis that a cluster of miRNAs, miR-3622a/b, at frequently deleted chr8p21 region are frequently lost in PCa leading to tumor progression.

A major challenge in PCa clinical management is posed by the lack of robust disease biomarkers for accurate diagnosis and prognosis [34–36]. Our analyses suggest that miR-3622b expression is widely attenuated in PCa clinical specimens and that miR-3622b expression is a promising PCa diagnostic biomarker that exhibits 100% specificity and 66% sensitivity. Interestingly, miR-3622b expression was inversely correlated with age-adjusted serum PSA values. In view of these results, we suggest miR-3622b as an alternative PCa diagnostic biomarker that can be used as a supplement to serum PSA testing. Our data also suggests that miR-3622b expression can be used to predict biochemical recurrence and low miR-3622b expression was found to be associated with poor biochemical recurrence-free survival. Though we did not observe a statistically significant correlation between low miR-3622b expression and pathological stage and tumor grade, miR-3622b expression was progressively lost in advanced tumors. In view of this, further studies with larger clinical cohorts are warranted to test the prognostic potential of miR-3622b in PCa. Overall, our study indicates that miR-3622b has significant potential as a diagnostic and prognostic PCa biomarker.

Restoration of miR-3622b expression in PCa cell lines led to reduced cellular viability, proliferation, invasiveness, migration and increased apoptosis. Collectively, these data support a tumor suppressive role for miR-3622b in PCa. In keeping with this, injection of miR-3622b mimics in established PCa mouse xenografts led to tumor regression *in vivo* suggesting that restoration of miR-3622b expression can be an attractive therapeutic modality in PCa. An anti-apoptotic role of miR-3622b in PCa was indicated by the induction of apoptosis and PARP cleavage following its overexpression.

Interestingly, our data suggests that miR-3622b directly represses EGFR in prostate cancer. EGFR, a tyrosine kinase receptor of the ERBB transmembrane growth factor receptor family, plays a key role in major cellular processes such as survival, proliferation, invasion [37–39]. It is frequently overexpressed in solid tumors from various organs, including PCa, causing tumor growth and progression and is an important therapeutic target [37–41]. EGFR expression has been reported to be significantly correlated with high tumor grade, advanced stage, high risk for PSA recurrence and shorter progression-free survival [40]. 100% of cases of metastatic hormone refractory PCa express EGFR. In our study, we discovered miR-3622b-mediated regulation of EGFR suggesting that this regulatory control may underlie the observed effects of miR-3622b overexpression on cell survival, proliferation and invasion. EGFR knockdown phenocopied the effects of miR-3622b overexpression in PCa cell line, lending support to our hypothesis. Targeting the EGFR axis is a potential therapeutic strategy in prostate cancer [42, 43]. The combination of EGFR inhibitor gefitinib and radiation has been reported to have promising activity against prostate cancer [43]. In view of our results, we propose that miR-3622b may be an important anti-cancer target for EGFR overexpressing prostate tumors.

In conclusion, our study shows that miR-3622b is frequently downregulated in PCa leading to upregulation of EGFR and culminating in effects on cell survival, proliferation, apoptosis and invasion. In view of our present results and our earlier study on miR-3622a, we propose that the miR-3622a/b cluster at frequently deleted chr8p21 locus plays important mechanistic roles in prostate cancer by regulating cardinal genes involved in tumorigenesis. Frequent loss of miR-3622a/b cluster at chr8p21 region causes downregulation of these genes leading to prostate cancer progression. Considering the association of chr8p21 loss with tumor progression and poor prognosis in prostate cancer [31, 32], our findings are highly significant as they support a novel concept that connects a long standing observation of frequent loss of a chromosomal region with a novel miRNA cluster in prostate cancer. Importantly, these miRNAs also have prognostic, diagnostic and therapeutic potential in the treatment of prostate cancer.

## MATERIALS AND METHODS

### Ethics statement

Investigation has been conducted in accordance with the ethical standards and according to the Declaration of Helsinki and according to national and international guidelines and has been approved by San Francisco Veteran Affairs Medical Center (SFVAMC) review board.

### Cell lines and cell culture

PCa cell lines (PC3, LNCaP, Du145) were obtained from the American Type Culture Collection (ATCC) and cultured under recommended conditions. PC3, LNCaP cell lines were maintained in RPMI 1640 media (UCSF cell culture facility) and Du145 cells were cultured in MEM media, each supplemented with 10% fetal bovine serum (FBS) (Atlanta biologicals) and 1% penicillin/streptomycin (UCSF cell culture facility). Immortalized non-transformed prostate epithelial cell line (BPH1) [44] was maintained in RPMI 1640 media supplemented with 5% FBS, and 1% penicillin/streptomycin. All cell lines were maintained in an incubator with a humidified atmosphere of 95% air and 5% CO2 at 37°C. Prostate cell lines were authenticated by DNA short-tandem repeat analysis. The experiments with cell lines were performed within 6 months of their procurement/resuscitation.

### miRNA/siRNA transfections

Cells were plated in growth medium without antibiotics ~24 hrs before transfections. Transient transfections of miRNA precursor (Ambion) or siRNA (Origene) was carried out by using Lipofectamine 2000 (Invitrogen) according to the manufacturer's protocol. For miRNA transfections, miR-3622b precursor (PM20243) or negative control (miR-CON) (AM17110) were purchased from Ambion. Trilencer-27 predesigned siRNA duplexes (SR301357) or universal scrambled negative control siRNA duplex (SR30004) purchased from Origene were used for siRNA-mediated *EGFR* knockdown. All miRNA/siRNA transfections were for 72h.

### Tissue samples

Formalin-fixed, paraffin-embedded (FFPE) PCa samples were obtained from the SFVAMC. Written informed consent was obtained from all patients and the study was approved by the UCSF Committee on Human Research. All slides were reviewed by a board certified pathologist for the identification of PCa foci as well as adjacent normal glandular epithelium.

### Laser capture microdissection (LCM)

Laser capture microdissection of tumor and adjacent normal areas was performed using the AutoPix System (Arcturus) as previously described [45, 46]. Briefly, 8μm sections were placed on glass slides, deparaffinized, stained with hematoxylin, dehydrated, and placed in the AutoPix instrument for microdissection. Areas of interest were captured with infrared laser pulses onto CapSure Macro LCM Caps.

### RNA and miRNA extraction

Total RNA was extracted from microdissected FFPE tissues using a miRNeasy FFPE Kit (Qiagen). A miRNeasy mini kit (Qiagen) was used for miRNA extraction from cultured cells and xenograft tumors following the manufacturer's instructions.

### Quantitative real-time PCR

Mature miRNAs and mRNAs were assayed using the TaqMan MicroRNA Assays and Gene Expression Assays, respectively, in accordance with the manufacturer's instructions (Applied Biosystems). Samples were normalized to RNU48 or GAPDH (Applied Biosystems) controls, as indicated. Taqman assays used were hsa-miR-3622b (assay ID 465068_mat), RNU48 (assay ID 001006), EGFR (Hs01076090_m1), GAPDH (Hs99999905_m1), The comparative Ct method was used to calculate the relative changes in gene expression on the 7500 Fast Real Time PCR System.

### Cell viability and clonogenicity assays

Cell viability was determined at 24, 48, 72 hours by using the CellTiter 96 AQueousOne Solution Cell Proliferation Assay Kit (Promega), according to the manufacturer's protocol. For clonogenicity assay, 48 hrs post-transfection, cells were counted, seeded at low density (1000 cells/plate) and allowed to grow until visible colonies appeared. Then, cells were stained with Giemsa and colonies were counted.

### Migration and invasion assays

Cell migration and invasion assay inserts (BD Biosciences) were used according to the manufacturer's protocol. Briefly, 48 hrs post-transfection, cells were counted and placed on control inserts (for migration) or matrigel inserts (for invasion) at 1x10^5^ cells/ml in serum-free medium and were allowed to migrate at 37°C for 24 h. After removing the cells from the top of the inserts, cells that migrated/invaded though the polycarbonate/basement membrane were fixed, stained and quantified at OD 560 nm after extraction.

### Apoptosis assay

Fluorescence-activated cell-sorting (FACS) analysis was done 72 hours post-transfection. The cells were harvested, washed with cold PBS and stained with 7-AAD and Annexin-V-FITC using a Annexin-V-FITC /7-AAD KIT (Beckman Coulter) for apoptosis analysis according to the manufacturer's protocol. Stained cells were immediately analyzed by FACS (Cell Lab Quanta SC; Beckman Coulter, Inc).

### Western blotting

Whole cell extracts were prepared in RIPA buffer [50 mmol/L Tris (pH 8.0), 150 mmol/L NaCl, 0.5% deoxycholate, 0.1% SDS, and 1.0% NP-40] containing protease inhibitor cocktail (Roche). Total protein was electrophoresed by SDS-PAGE and Western blotting was carried out according to standard protocols. The following antibodies were used for Western blotting: EGFR (Cell Signaling, cat no. 4267), GAPDH (Santa Cruz Biotechnology, sc-32233).

### Luciferase assays

*EGFR* 3'UTR region containing potential target sequences complementary to the miR-3622b seed sequence were cloned downstream of the luciferase gene in the pmiRGLO luciferase vector (Promega) according to the manufacturer's instructions. Mutated 3'UTR sequences complementary to miR-3622b were cloned in the same vector. Primers used for these clonings were synthesized by Invitrogen and are listed in Supplementary Table S1. 3'-UTR *EGFR* reporter/ control constructs (0.2 ug each) in the pmiRGLO luciferase vector (Promega) were each cotransfected in PC3 cells with 50 nM miR-CON/ miR-3622b precursor (Ambion) using Lipofectamine 2000 (Invitrogen). Firefly and Renilla luciferase activities were measured by using the dual luciferase reporter assay system (Promega) 48 hr post-transfection in accordance with the manufacturer's protocol. Firefly luciferase was normalized to Renilla luciferase activity.

### *In vivo* intratumoral delivery of miR-3622b

All animal care was in accordance with the guidelines of the SFVAMC and the study was approved by the San Francisco VA IACUC. The therapeutic potential of miR-3622b was examined by local administration in established tumors in a PCa xenograft mouse model as previously described [45, 47, 48]. Nude mice (5 weeks-old, Simonsen Laboratories) (n=12) were injected with 2 X 10^6^ PC3 cells (in 100 μl volume) subcutaneously in the right flanks. Once palpable tumors developed, caliper measurements were taken twice a week and tumor volumes were calculated as x^2^y/2, where width (x) < length (y). Synthetic miR-3622b precursor/ miR-CON (6.25 μg each) complexed with 1.6 μL siPORTamine transfection reagent (Ambion) in a volume of 50 μL PBS was delivered intratumorally every 4 days. Synthetic miRNAs are double-stranded, ready-to-use miRNA precursors and were procured from Ambion (pre-miR, cat. no. AM17100). Mice were killed 2 days after the last treatment (day 56) and tumors were harvested.

### Statistics

All quantified data represents an average of triplicate samples or as indicated. Data are represented as mean ± S.E.M or as indicated. Two-tailed Student's t-test was used for comparisons between groups. All statistical analyses were performed using MedCalc version 10.3.2. Results were considered statistically significant at P ≤ 0.05. The Wilcoxon Signed Rank test was used to assess the difference between miR-3622b expression in clinical tissues (tumor and normal adjacent). Correlations between miR-3622b expression and clinicopathological parameters were assessed using Chi squared test. For Kaplan-Meier survival analysis, cases were stratified into high (relative expression > 1.25)/no change (relative expression 0.75-1.25) and low expression groups (relative expression <0.75) based on miR-3622b expression levels. Receiver operating characteristic (ROC) curves were calculated to determine the potential of miR-3622b to discriminate between malignant and normal samples. miR-3622b is modeled as a categorical variable (no change, low expression, high expression). Areas under the ROC curve (AUC) were estimated and reported with 95% DeLong Confidence Intervals (CI). The discriminatory ability of miR-3622b expression was further characterized based on a dichotomous variable (no change/ high expression vs. low expression); using apparent prevalence, true prevalence, sensitivity, specificity, positive predictive value, negative predictive value, positive likelihood ratio and negative likelihood ratio.

## SUPPLEMENTAL FIGURES AND TABLE



## Abbreviations

PCa: Prostate cancer
miRNA: MicroRNA
CNAs: Copy Number Alterations
3′UTR: 3′Untranslated Region
NKX3.1: NK3 homeobox 1 gene
EMT: Epithelial-to-Mesenchymal Transition
ZEB1: Zinc Finger E-Box Binding Homeobox 1
SNAI2: Snail Family Zinc Finger 2
TCGA: The Cancer Genome Atlas
BPH1: Benign Prostatic Hyperplasia epithelial cell line
PSA: Prostate Specific Antigen
EGFR: Epidermal Growth Factor Receptor
ERBB: Erb-b2 receptor tyrosine kinases
BMI1: BMI1 Proto-Oncogene; Polycomb Ring Finger
7-AAD: 7-Aminoactinomycin D
FFPE: Formalin-fixed, paraffin-embedded
LCM: Laser Capture Microdissection
T stage: Tumor stage
N stage: Lymph node metastasis
M stage: distant metastasis
S.E.M: Standard Error Mean
ROC: Receiver operating characteristic
AUC: Area under the ROC curve
