# Supplementary material for: Novel tumor suppressor microRNA at frequently deleted chromosomal region 8p21 regulates Epidermal Growth Factor Receptor in prostate cancer
Source: Oncotarget. 2016 Sep 6;7(43):70388–403. doi: 10.18632/oncotarget.11865 (PMC5342560; doi:10.18632/oncotarget.11865)
Supplement: Supplementary file 1 [file oncotarget-07-70388-s001.pdf]

Novel tumor suppressor microRNA at frequently deleted chromosomal region 8p21 regulates Epidermal Growth Factor Receptor in prostate cancer

SUPPLEMENTAL FIGURES AND TABLE

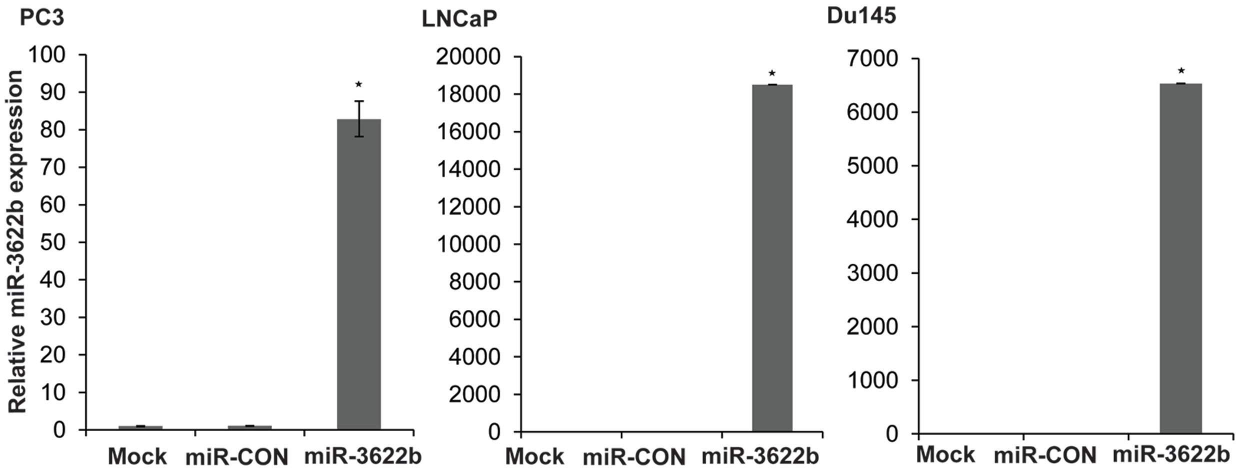

Supplementary Figure S1: miR-3622b overexpression in prostate cancer cell lines. Relative miR-3622b expression in PC3/LNCaP/DU145 cells transfected with miR-CON/miR-3622b as assessed by real-time PCR. Data were normalized to RNU48 control.

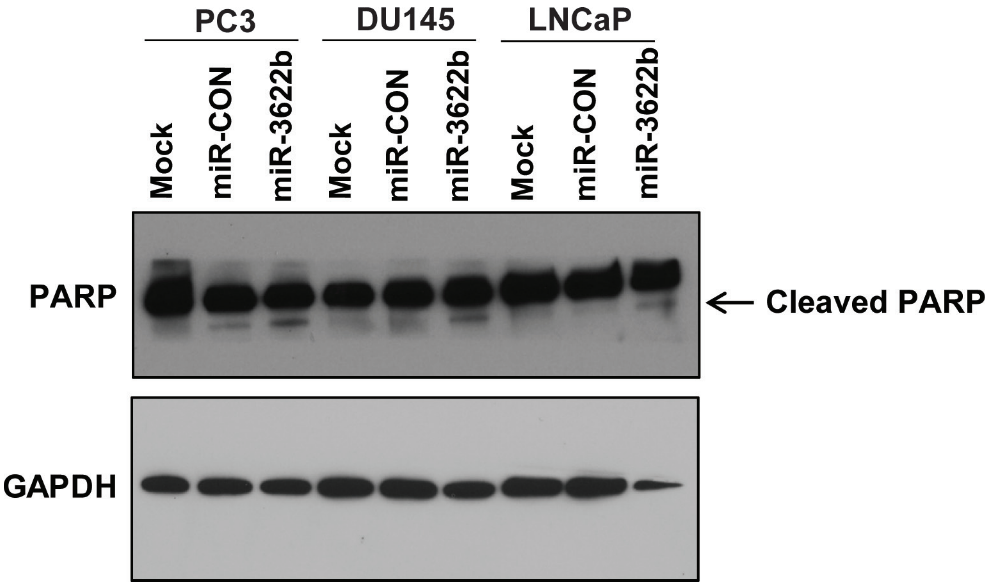

Supplementary Figure S2: miR-3622b overexpression induces PARP cleavage in prostate cancer cell lines. Immunoblots of endogenous PARP in PC3 (left panels), Du145 (middle panels) and LNCaP cells (right panels) transfected with mock/miR-CON/miR-3622b. GAPDH was used a loading control. Cleaved PARP was increased upon miR-3622b transfection as compared to controls.

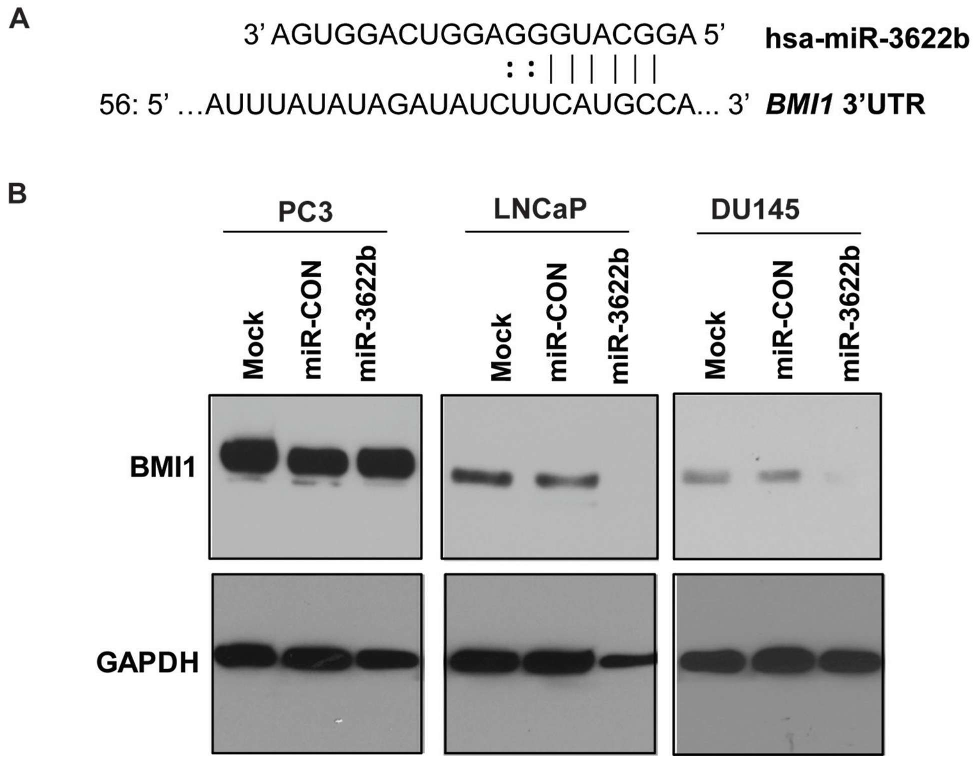

Supplementary Figure S3: miR-3622b targets Bmi1 in prostate cancer cell lines (related to Figure 7A). A. Schematic representation of BMI1 3' UTR showing potential miR-3622b binding site. **B.** Representation of BMI1 blot shown in Figure 7A. BMI1 blot is shown with the corresponding GAPDH controls (corresponding GAPDH for BMI1 was omitted due to space constraints). Immunoblots of endogenous BMI1 in PC3 (left panels), LNCaP (middle panels) and Du145 cells (right panels) transfected with mock/miR-CON/miR-3622b. GAPDH was used a loading control.

Supplementary Table S1: List of primers used for *EGFR* 3' UTR cloning and mutagenesis

Primer	Sequence (5'-3')
EGFR-1- sense	AAACTAGCGGCCGCTAGT CCTCCATCCCAACAGCCATGCCCT
EGFR-1-anti sense	CTAGAGGGCATGGCTGTTGGGATGGAGGACTAGCGGCCGCTAGTTT
EGFR-1- mismatch sense	AAACTAGCGGCCGCTAGTCCTCCATCCCAACAGGCTTCGGCT
EGFR-1-mismatch anti sense	CTAGAGCCGAAGCCTGTTGGGATGGAGGACTAGCGGCCGCTAGTTT
EGFR-2- sense	AAACTAGCGGCCGCTAGTGCTCCTCTAATTACACCATGCCCT
EGFR-2-antisense	CTAGAGGGCATGGTGTAATTAGAGGAGCACTAGCGGCCGCTAGTTT
EGFR-2- mismatch-sense	AAACTAGCGGCCGCTAGTGCTCCTCTAATTACAGCTTCGGCT
EGFR-2- mismatch-antisense	CTAGA GCCGAAGCTGTAATTAGAGGAGC ACTAGCGGCCGCTAGTTT
EGFR-3- sense	AAACTAGCGGCCGCTAGTAGAAAAATCCAGTTGCATGCCATT
EGFR-3- antisense	CTAGAATGGCATGCAACTGGATTTTTCTACTAGCGGCCGCTAGTTT
EGFR-3- mismatch-sense	AAACTAGCGGCCGCTAGTAGAAAAATCCAGTTGGAACCGATT
EGFR-3- mismatch-antisense	CTAGAATCGGTTCCAACTGGATTTTTCTACTAGCGGCCGCTAGTTT

EGFR 3'UTR region containing target sequences complementary to the miR-3622b seed sequence were cloned downstream of the luciferase gene in the pmiRGLO luciferase vector (Promega). The 3' UTR region of *EGFR* possess three potential miR-3622b binding sites (Figure 7B). Sites 1 and 2 are 7mer-m8 while site 3 is 7mer-A1. Mutated 3'UTR sequences complementary to miR-3622b (represented in Figure 7B) were cloned in the same vector. The primers used for clonings were synthesized from Invitrogen and are listed in the table above.
